# The NADP^+^-dependent IDH1 mutation and its relevance for glioblastoma patient survival

**DOI:** 10.1186/2049-3002-2-S1-P79

**Published:** 2014-05-28

**Authors:** Cornelis JF Van Noorden

**Affiliations:** 1 Department of Cell Biology and Histology, Academic Medical Center, University of Amsterdam, 1105 AZ Amsterdam, The Netherlands

## 

The metabolic consequences of the NADP^+^- dependent isocitrate dehydrogenase (IDH) 1 mutation was studied quantitatively in glioblastoma, the most aggressive form of brain tumor. The mutation is found in 70-80% of secondary glioblastoma. Metabolic rewiring of cells due to the mutation causes gliomagenesis by the production of 2-hydroxyglutarate. On the other hand, glioblastoma patients with the IDH1 mutation have a significant survival benefit of approx. one year. We found that the NADPH production capacity in glioblastoma is reduced by 38% when IDH1 is mutated [1]. We hypothesized that this reduced production capacity of NADPH, a major metabolite involved in detoxification processes, renders glioblastoma cells less resistant against irradiation and chemotherapy and thus prolong patient survival [2]. We also demonstrated that cellular metabolism is rewired in IDH1-mutated glioblastoma cells by elevated mitochondrial activity [3]. Further metabolic studies of human and rodent tissues showed that IDH activity is responsible for 65% of the NADPH production capacity in human brain and only for 15% in rodent brain whereas the pentose phosphate pathway was responsible for 85% of the NADPH production capacity in rodent tissues [4]. It explains why the pentose phosphate pathway was always linked with cancer and not IDH because most studies on metabolism and cancer have been performed in rodents. In conclusion, our metabolic study strongly suggests that the reduced NADPH production capacity in IDH1-mutated gliomablastoma makes cancer cells less resistant and as a consequence is at least partly responsible for prolonged patient survival.
